# Not All Diabetic Ketoacidosis in Infant Is Type 1: A Case Report Permanent Neonatal Diabetes

**DOI:** 10.1016/j.aace.2023.10.004

**Published:** 2023-10-17

**Authors:** Doua Khalid Al Homyani, Lina Al Homaiani

**Affiliations:** 1 Pediatric Endocrine in Taif Children Hospital, Taif, Saudi Arabia; 2 IBN Sina National College For Medical Studies, Jeddah, Saudi Arabia

**Keywords:** Neonatal diabetes, *INS* gene, Diabetic Ketoacidosis

## Abstract

**Background/Objective:**

Neonatal diabetes is a monogenic type of diabetes mellitus. It arises at the first 6 months of age and can be classified as permanent or transient. There are limited cases of neonates with DKA who have heterozygous mutations in *INS* and PKHD1 genes, especially in Saudi Arabia. We present a case of neonatal diabetes with diabetic ketoacidosis (DKA) born to consanguineous parents in Saudi Arabia. This study aims to highlight the importance of the genetic mutations associated with neonatal diabetes and identify the clinical manifestation features of neonatal diabetes.

**Case Report:**

A six-month-old boy born to consanguineous parents with a family history of neonatal diabetes was diagnosed with DKA. The case was presented to the emergency department (ED) with vomiting and increased urination for 3 days. The child showed signs of severe dehydration and severe metabolic acidosis with a high anion gap and elevated hemoglobin A1C level (16.3%) was reported. According to the genetic test, the patient had an *INS* and *PKHD1*gene mutation. The treatment was initiated according to the DKA protocol, and then he received subcutaneous insulin.

**Discussion:**

Neonatal diabetes is a condition caused by several gene mutations. In this case, heterozygous mutations in *INS* and PKHD1 genes were reported. The type of gene mutation could predict neonatal diabetes type, whether permanent or transient, and its response to treatment.

**Conclusion:**

Genetic testing for neonates soon after birth is suggested for the early detection and classification of neonatal diabetes, especially among children with a family history of neonatal diabetes.

**Highlights**

• NDM is hyperglycemia within the first 6 months up to 12 months of life.
• Very early onset diabetes mellitus seems to be unrelated to autoimmunity in most instances.
• Gathered the probands’ family history carrying relevant mutations.
• Genetic testing is important and can significantly alter the treatment and outcome.

**Clinical Relevance**

This case highlights the clinicians to consider a neonatal form of diabetes if diagnosis is made up within 6 months of life especially for infants born to consanguineous families. We report the heterozygous mutation in INS 326G>A (p.C109Y). So, immediate referral for genetic testing after a clinical diagnosis of neonatal diabetes is important.

## Introduction

Diabetes mellitus (DM) is a group of metabolic disorders characterized by hyperglycemia. It is one of the most common endocrine disorders worldwide. However, neonatal diabetes is considered a rare type of DM that develops within the first weeks or 6 months of life.^1^, ^2^, ^3^

Neonatal diabetes can be classified into transient and permanent neonatal diabetes mellitus (PNDM). Transient neonatal diabetes mellitus (TNDM) is an insulin production developmental condition associated with intrauterine growth retardation, and 50% to 60% of cases resolve within a year. PNDM is a less common form in which lifelong exogenous insulin therapy is required.^1^ The approximate incidence of neonatal diabetes is 1 in 90 000 to 160 000 live births worldwide and 1 in 21 196 live births in Saudi Arabia.^1^^,^^3^

Previous researchers have found that neonatal diabetes can be due to a monogenic disorder or impaired pancreatic development. Different gene mutation types are involved in the pathogenesis of neonatal diabetes. Gene abnormalities were found in *KCNJ11* and *ABCC8* genes affecting potassium channels of beta-cells, *INS* gene affecting insulin production, and chromosome *6q24*.^1^, ^2^, ^3^

The literature contains limited cases of neonates with diabetic ketoacidosis (DKA) caused by heterozygous mutations in *INS* and PKHD1 genes, especially in Saudi Arabia. Here, we present a case of neonatal diabetes in Saudi Arabia with DKA caused by heterozygous mutations in *INS* 326G>A (p.C109Y) and PKHD1 genes. It also highlights the importance of genetic testing in infants born to consanguineous parents with a family history of neonatal diabetes.

## Case Report

A six-month-old boy was admitted to the emergency department (ED) at Children’s Hospital in Taif due to vomiting and increased urine output for 3 days. His mother was 23 years of age when he was born, and he was considered healthy at birth. He was full-term born by Lower Segment Cesarian Section to consanguineous parents (second-degree). The birth weight was borderline normal, 2.5 Kg. The postnatal period was uneventful, and the parents did not observe any conditions that occurred among patients with neonatal diabetes.

At the examination, the child showed signs of severe dehydration and had Kussmaul breathing. His temperature was 37.6 °C, heart rate was 155 bpm, respiratory rate was 60 rpm, and blood pressure was 88/57 mmHg. His weight was 7 kg (10-25th percentile) and length 64 cm (third percentile). He had no dysmorphic features. His abdomen was soft, not distended, and he had no organomegaly. The genitourinary examination showed bilateral descended testicles.

Ultrasound revealed structurally normal pancreas and kidneys. Cardiovascular, respiratory, and neurologic examinations were unremarkable.

Laboratory investigations revealed severe metabolic acidosis with a high anion gap. Venous blood gas was PH 7.0, HCO3 6.6, and base deficit was 24. Complete blood counts were normal, and renal panel and liver function tests were normal in Table. Urinary ketones and glucose were positive +3, Random Blood Sugar was 30 mmol/L, and hemoglobin A1C was 16.3% (reference range: below 5.7%). Glutamic acid decarboxylase-65 antibody and insulinoma-associated antibody were negative; the fasting C-peptide was 0.7 ng/mL (reference range: 1-3 ng/mL) Table.

**Table:** Lab Results: Liver Function Test, Renal Function Test, and C-Peptide Level

Test	Result	Reference range
AST	15.8	0-41 U/L
ALT	16.3	0-41 U/L
GGT	24	8-61 U/L
ALBUMIN	44.6	38-54 G/L
BUN	5.4	2.6-8.07 mmol/L
CREATININE	23	15-37 ummol/L
C-PEPTIDE	0.7	1-3 ng/ml

The patient was diagnosed with DKA. The treatment was initiated according to DKA protocol which aim to normalize fluid-volume status, hyperglycemia, electrolytes, and ketoacidosis. Subsequently, intravenous insulin was changed to subcutaneous insulin.

The pedigree of the neonate is shown in Figure, the mother had a twin sister, and both she and her twin sister developed diabetes mellitus at 3 months of age and are on insulin. The twin sister was thought to be identical, but no studies relating to this were performed.

**Fig:**
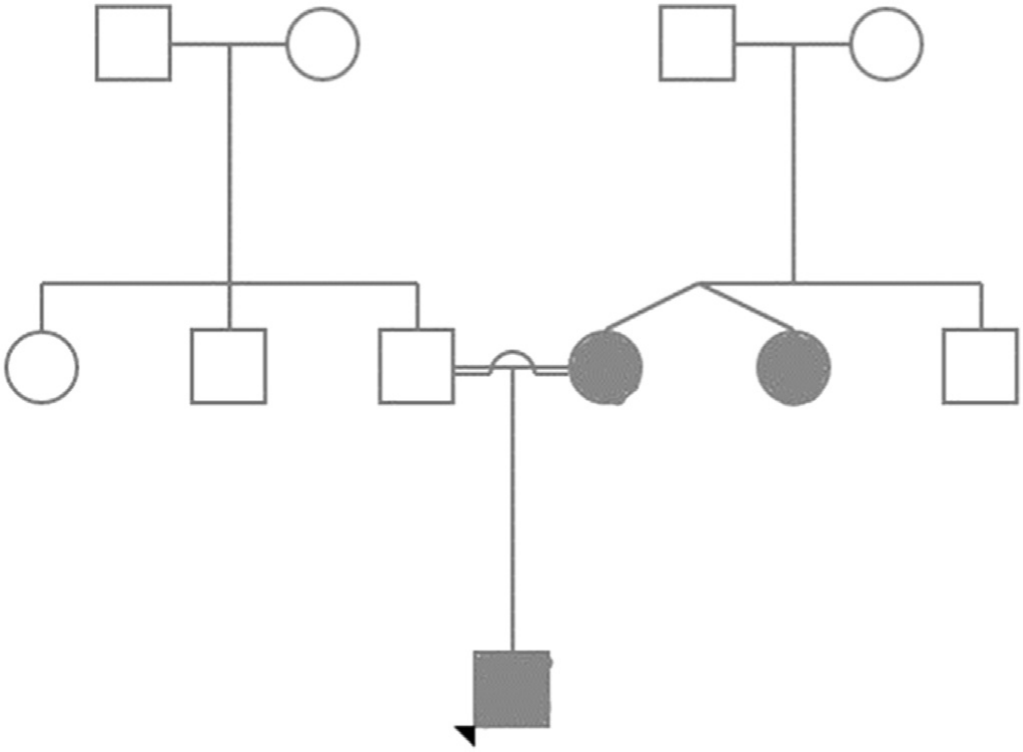
Pedigree Chart: “Males are represented as squares; females are represented as circles. Shaded symbols stand for an individual who is affected by the proband’s condition; unshaded symbols mean they are not affected by the neonate’s condition. A horizontal line between male and female symbols indicates mating, and the resultant children of each mating are shown by a descending line with offshoots to the line for more than one offspring. Two diagonal lines joining together in the figure point to Identical twin siblings.”

However, the father had normal fasting blood glucose levels.

The genetic test Whole exome sequencing (WES) was carried out by blood sample at research lab and revealed a heterozygous mutation in *INS* 326G>A (p.C109Y) and a heterozygous mutation in PKHD1 which was reported at the same moment. Efforts to obtain genetic tests of the neonates’ parents and aunt were made but were not successful.

Presently, the patient is on long-acting insulin and short-acting insulin. His most recent HbA1c level is 9.4%, and his current weight is 9.5 kg.

## Discussion

Neonatal diabetes is a very rare condition that arises in the first 6 months of life and rarely between 6 months and 1 year. It may present with hyperglycemia, severe dehydration, and failure to thrive.^4^ In our case, the patient was a six-month-old boy with a fever, severe dehydration, severe metabolic acidosis, and high HbA1C, and he was diagnosed with DKA.

Diagnosis of neonatal diabetes is challenging. The reports suggest that neonatal diabetes may mimic sepsis in symptoms.^5^ About 20 gene mutation types are involved in the pathogenesis of neonatal diabetes. These genes include, PLAGL1 HYMAI (6q24), ZFP57, KCNJ11, ABCC8, INS, EIF2AK3, FOXP3, GCK, PDX1, NEUROD1, NEUROG3, RFX6, IER3IP1, HNF1B, GLIS3, GATA6, PAX6, WFS1, SLC19A2, and SLC2A2. It is estimated that the most common forms of neonatal diabetes are caused by mutations in KCNJ11, INS, 6q24, and ABCC8. On the other hand, GATA6, PDX1, EIF2AK3, and FOXP3 genes are among the least common genes attributing to DM among neonates.^3^

Recently, heterozygous coding mutations in the *INS* gene that encodes preproinsulin were indicated to be an essential cause of PNDM. These mutations eliminate the normal folding of proinsulin, which causes the death of beta-cells through endoplasmic reticulum stress and apoptosis. Recessive or dominant coding mutations in the *INS* gene could be found among patients with neonatal diabetes.^6^

Approximately more than 90 different dominant mutations in the *INS* gene have been detected in probands with PNDM. On the other side, only 25 different homozygous or compound heterozygous mutations in the *INS* gene have been reported in probands with PNDM and TNDM.^7^

In this report, the genetic test has shown heterozygous mutations in *INS* 326G>A (p.C109Y) and *PKHD1* genes. The PKHD1 leads to autosomal recessive polycystic kidney disease.^8^ According to a previous study, neonatal diabetes can be caused by a mutation of 21 genes, the most common gene mutations were *ABCC8* and *KCNJ11* potassium channel subunit genes reported.^4^ Moreover, heterozygous mutations in the *INS* gene in some cases and less frequently homozygous mutations in the *INS* gene were also reported. Another report mentioned that heterozygous autosomal dominantly inherited mutations in the *INS* gene are the second most common cause of permanent neonatal diabetes (after mutations in KCNJ11).^9^ Other types of gene mutations were observed among cases with neonatal diabetes. These mutations include a heterozygous missense mutation in the *GATA6* gene ^3^ and a *PDX1* gene mutation.^10^

Recessive INS mutations have atypical clinical manifestations, including marked low birth weight, early-onset diabetes (median 1 week), insulin deficiency, ketoacidosis, and absence of extrapancreatic features.^3^ According to the current case, the child was diagnosed with DKA, which is one of the clinical manifestations of INS mutations.

The type of gene mutation can predict the type of neonatal diabetes, whether permanent or transient, and its response to sulfonylurea treatment.^4^ Some mutations may contribute to TNDM or PNDM, such as *ABCC8*, *KCNJ11*, and *INS* gene mutation. Meanwhile, other mutations contribute to only one type of neonatal diabetes, for instance, chromosome *6q24* mutation leads to TNDM, and *GATA6* gene mutation causes PNDM.^4^^,^^9^ In our report, the prognosis of this case is not well-defined as the patient is only 6 months old and on regular insulin.

Reports of increased neonatal diabetes when parental consanguinity. The parents in our report are consanguineous. Moreover, the patient has a family history of neonatal diabetes; the mother and her twin sister were diagnosed at the age of 3 months. A previous study reported that most patients born to consanguineous parents had the mutation presented in recessive genes. On the other hand, dominant gene mutations were responsible for most nonconsanguineous family cases.^11^

Genetic testing and next-generation sequencing have proved efficacy in identifying more than 80% of neonatal diabetes cases. Early detection can enhance the treatment choice and the predictability of associated conditions.^4^ Moreover, it can lead to a good prognosis prediction and assessment of the type of neonatal diabetes. Thus, it is recommended to perform genetic testing for all suspected cases, especially in the affected families of diabetes under 12 months of age.^3^^,^^10^

## Conclusion

This case highlights the clinicians to consider a neonatal form of diabetes if the diagnosis is made up within 6 months of life, especially for infants born to consanguineous families. In addition, genetic testing is recommended among all cases of neonatal diabetes and their parents, especially neonates born to consanguineous parents.

## Disclosure

The authors have no multiplicity of interest to disclose.
